# “Excited When They See Their Name in Print”: Research Outputs from an Australian Medical Program

**DOI:** 10.1007/s40670-024-02029-5

**Published:** 2024-03-29

**Authors:** Harry Hieu Dinh, Kerry Uebel, Maha Pervaz Iqbal, Ari Grant, Boaz Shulruf, Sally Nathan, Khanh Vo, Greg Smith, Jane Ellen Carland

**Affiliations:** 1 School of Population Health, Faculty of Medicine and Health, UNSW Sydney, Kensington, Australia; 2 Faculty of Medicine and Health, UNSW Sydney, Kensington, Australia; 3 Centre for Health Systems and Safety Research, Australian Institute of Health Innovation, Sydney, Australia; 4 Faculty of Medicine, Health and Human Sciences, Macquarie University, Sydney, Australia; 5 UNSW Library, UNSW Sydney, Kensington, Australia; 6 School of Biomedical Sciences, Faculty of Medicine and Health, UNSW Sydney, Kensington, Australia; 7 Department of Clinical Pharmacology and Toxicology, St Vincent’s Hospital, Sydney, Darlinghurst, Australia; 8 School of Clinical Medicine, St Vincent’s Healthcare Clinical Campus, Faculty of Medicine and Health, UNSW Sydney, Kensington, Australia

**Keywords:** Medical student, Student research, Research output, Student publication, Medical education, Supervisor support

## Abstract

To promote evidence-based practice, medical schools offer students opportunities to undertake either elective or mandatory research projects. One important measure of the research program success is student publication rates. In 2006, UNSW Medicine implemented a mandatory research program in the 4th year of the undergraduate medical education program. This study identified student publication rates and explored student and supervisor experiences with the publication process.

A retrospective audit of student publications from the 2007, 2011, and 2015 cohorts was undertaken to look at trends over time. Data collected included type of publication and study methodology. Semi-structured interviews were conducted with a sample of undergraduate students (*n* = 11), medical graduates (*n* = 14), and supervisors (*n* = 25) and analysed thematically.

Student publication rates increased significantly (*P* = 0.002) from 28% in 2007 to 50.2% in 2015. Students able to negotiate their own project were more likely to publish (*P* = 0.02). Students reported personal affirmation and development of research skills from publishing their research findings, while graduates noted improved career opportunities. Supervisors expected students to publish but identified the time to publications and student motivation as key factors in achieving publication(s).

A high publication rate is possible in a mandatory research program where students can negotiate their own topic and are given protected time. Publications happen after the research project has finished. Critical factors in successful publication include supervisor support and student motivation. Given the importance of the supervisor’s role, staff development and faculty support to train and develop a body of skilled supervisors is required.

## Introduction

Incorporation of research programs within medical school curricula is recommended to enable future doctors to practice evidence-based medicine [1] and participate in future research during their careers. Evidence-based medicine requires doctors to be able to understand and apply outcomes of clinical research in their practice. Studies show that participation of students in research during their medical degree helps them to develop the necessary research skills and attributes [1], including critical reading and writing skills, as well as understanding research methodologies [2] and the ability to interpret clinical data [3]. Participation in research projects also helps medical students develop other important skills including self-directed learning and communication [1] and provides opportunities for mentoring, social networking, and teamwork skills [3]. Beyond skill development, research exposure has also been shown to influence the future careers of students. Involvement in research has been shown to motivate students to pursue academic careers [4] and to be more interested in research later in their careers [5].

Different approaches have been used by medical programs to facilitate medical student involvement in research [1, 5–8]. Some medical schools offer elective research programs, including access to summer research electives [7, 9, 10] and intercalated research degrees [11, 12]. Other medical schools have incorporated mandatory research programs that consist of curricular components [13], graduation projects or theses [14]. Mandatory research projects can be completed longitudinally, alongside teaching [15], or during a dedicated research block that allows students to focus on their project. Although students participating in both elective and mandatory programs report benefits associated with research exposure, several difficulties are also noted, including time away from clinical teaching [16](in the case of dedicated research blocks), time pressures [15] (in the case of longitudinal research programs), and the incurring of extra university fees [2, 17].

Since 2006, the 6-year undergraduate degree at UNSW Medicine has mandated 4th year students to complete a research project under the supervision of a senior research mentor during a 9-month research block in order to better prepare them to practice evidence-based medicine [18]. Given the concerns of some students about mandatory research [16, 17], and some evidence that mandatory research programs have a lower publication rate than voluntary programs [5], this study aimed to document publication rates and to identify factors that promote publication for medical student research projects of the UNSW research program within three cohorts between 2007 and 2015.

## Methods

This study used a mixed-method approach, which includes a retrospective audit of student publications arising from mandatory research projects and semi-structured interviews with a sample of students, graduates, and supervisors.

### Context

Students in the 4th year of the UNSW medical program undertake a mandatory research project, referred to as an Independent Learning Project (ILP). ILP students are required to commit 25 h each week to their research for 30 weeks. High-achieving students can undertake a Bachelor of Science (Medicine) Honour’s degree which requires 35 h each week over 34 weeks. In the 3rd year of their degree, students are encouraged to approach prospective supervisors to negotiate their research project across a period of 6 months. They are supported by the Faculty, with guidance from the year 4 academic coordinator and administrative staff. For students who are not able to successfully negotiate a research project (negotiated project), the medicine faculty allocates a project and a supervisor (allocated project). Students are required to do an oral presentation of their work and submit a written research manuscript to the Faculty on completion of their ILP/Honours.

### Retrospective Audit

A retrospective audit of student publications from three cohorts—2007, 2011, and 2015—was undertaken to document changes as the program matured. We sampled these three cohorts to make the search more manageable, to provide time for embedding of the program in 2006 and to allow for sufficient time for production of publications for 5 years after each cohort. Student and supervisor names, project topics, and whether projects were negotiated or allocated were provided by UNSW Medicine administration. A search using names of the student and primary supervisor (in combination) from year of project to 5 years after project completion (2007–2012, 2011–2016, and 2015–2020) was undertaken on the following databases: Scopus, Web of Science, Embase, CINAHL, and Informit. Publications identified were screened and included if both student and supervisor were listed as authors and the title aligned with the project topic. Inclusion of publications was confirmed by consensus at weekly meetings of the research team.

Data collected included title, journal name, article title, number of authors, student first author (yes/no), type of publication (original article, reviews, conference abstracts, or other), year of publication, metrics (Google scholar citation count, Scimago JR journal quartile), and research methodology. Google Scholar citation counts were collected as every publication identified was indexed in this search tool. Citation count was normalised by dividing by the number of years since publication. Scimago JR was used to determine the quartile for each journal in the year of publishing. When more than one quartile ranking was listed, the highest ranked quartile was recorded.

### Statistical Analysis

The primary explanatory variables were the year of project completion and negotiated or allocated research project. The main categorical outcome was the proportion of students who had publications from their ILP/Honour’s project. Other outcomes included the publication type, year of publication, proportion of the publication outputs with student first authors, citation count, methodology of study in publication and journal quartile.

Categorical variables were described as frequencies and percentages. Cross-tabulation using Kendall’s tau B test was used to compare two categorical ordinal variables and determine significance. The number of publications for each cohort and student, treated as a continuous variable, was skewed in nature. Thus, non-parametric statistical methods were reported. To determine significance between two categorical variables the Mann–Whitney *U* test was used. The Kruskal–Wallis test was used for more than two categorical variables. Pairwise comparisons were conducted in the event of an overall significant result. Simple descriptive analysis was conducted using Excel spreadsheet, and statistical analyses were conducted by IBM SPSS statistical software version 22 [19].

### Semi-Structured Interviews

Interviews were conducted between June 2020 and March 2021 with 6th year medical students, recent medical graduates, and supervisors to capture overall experience of the ILP/Honours program. Convenience sampling was used for participant recruitment. Broadcast emails inviting people to participate were sent to all 6th year students (November 2020; response rate = 11/290), graduates from 2015–2019 student cohorts (May 2020; response rate = 14/1218), and current supervisors (March 2021; response rate = 25/206). Phone interviews were conducted by members of the research team. They were audiotaped and transcribed verbatim.

### Qualitative Data Analysis

De-identified transcripts were analysed independently by two researchers using an inductive approach. Transcripts were initially read to identify components of the interviews that focussed on publications and presentations. NVivo 12 was used to aid a thematic analysis to develop relevant themes. The major themes were reviewed and revised by the research team across a series of meetings.

## Results

### Retrospective Audit

The number of students enrolled in the ILP/Honours program varied across the three student cohorts with 206 students enrolled in 2007, 262 students in 2011, and 205 students in 2015. Across the three cohorts, the proportion of students successfully negotiating their own projects increased significantly (*P* < 0.0001) from 56.8% (117/206) in 2007 up to 95.1% (195/205) in 2015 (Table 1).

**Table 1:** Characteristics of student cohorts and publications produced by ILP/Honours Medicine students in 2007, 2011, and 2015

	**Student cohort**		
	**2007**	**2011**	**2015**
**Number of students**	206	262	205
**Negotiated projects**	117 (56.8%)	224 (85.4%)	195 (95.1%)
**Total publications**	147	255	232
**Publication rate**^**a**^	28.2% (58/206)	44.7% (117/262)	50.2% (103/205)
**Student 1st authorship**^**b**^	43.5% (64/147)	44.6% (111/255)	49.4% (114/232)
**Publication type**			
*** Original article***	46.3% (68/147)	37.3% (95/255)	50.0% (116/232)
*** Review***	13.6% (20/147)	5.9% (15/255)	8.2% (19/232)
*** Conference abstract***	35.4% (52/147)	47.5% (121/255)	34.5% (80/232)
*** Others***	4.8% (7/147)	9.4% (24/255)	7.3% (17/232)
**Methodology**^**c**^** (%)**			
*** Quantitative***	69.5% (66/95)	70.2% (92/131)	77.0% (117/152)
*** Qualitative***	7.4% (7/95)	6.9% (9/131)	7.2% (11/152)
*** Others***^***d***^	23.2% (22/95)	22.9% (30/131)	15.8% (24/152)

^a^Percentage of students who produced a publication in each cohort, as a proportion of total number of students in each cohort (2007, *N* = 206; 2011, *N* = 262; 2015, *N* = 205)

^b^Proportion of publications with the student being 1st author, as a proportion of total publications identified for each cohort (2007, *N* = 147; 2011, *N* = 255; 2015, *N* = 232)

^c^Excludes conference abstracts

^d^Others include mixed methodology, reviews, abstracts, letters to the editor, book chapters, case report/series

### Research Productivity

The retrospective audit identified 585 research outputs in total across the three student cohorts (147 in 2007, 255 in 2011, and 232 in 2015), with a median of 1 (range, 1–24) publication identified for students who published. Most publications were original articles (279) or conference abstracts (253) (Table 1).

The proportion of students who published increased significantly over time from 28.2% (58/206) in 2007 up to 50.2% (103/205) by 2015 (*P* = 0.002) (Table 1). Students who negotiated their own projects had a significantly higher publication rate than those allocated a project (*P* = 0.02). Students were the first author of almost half of all publications identified (Table 1). Most of the published manuscripts (excluding abstracts where methodology was not always clear) reported on studies that used quantitative methods (Table 1). A median lag time from completion of the 4th year to publication was 2 years (range 0–5 years), noting publications beyond 5 years of project completion were not documented.

### Quality of Publications

The median number of citations for each publication was 32 (range 0–2272) in 2007, 23 (range 0–225) in 2011, and 9 (range 0–118) in 2015. When citation counts were normalised by years since publication, there was no significant difference between cohorts. More than half of the publications identified for each student cohort were in Q1 journals (2007: 79/142, 55.6%; 2011: 167/242, 67.9%; 2015: 138/222, 62.2%), with no significant change in journal quartile distribution across the three cohorts.

### Semi-Structured Interviews

Of the interview participants, 6 of the 11 students and 8 of the 14 graduates reported that they had presented their ILP/Honours project findings at a conference or had published in a peer-reviewed journal. The rest had not published their findings. The supervisors we interviewed had a wide range of experience with ILP/Honours supervision from one student to over 20 students, and they reported variable student publication success. Three key themes were identified; publication was a desirable outcome, publication developed skills and improved career opportunities, and supervisor support and student motivation impacted publication success.

#### Publication Was a Desirable Outcome

Although producing a publication was not a requirement of the program, most supervisors expected and encouraged students to publish their findings. Supervisors reported telling their students that it “…is an expectation, you will get a publication out of this [research project]” (Supervisor 3). Some students and graduates discussed the personal affirmation they gained from producing research outputs. One student commented “…[I] feel that [my] findings have been listened to and appreciated” (Student 9). One supervisor described that students were “…excited when they see their name in print for the first time…” (Supervisor 1). Conversely, students who did not produce a research output were disappointed; “I didn't get anything out of this… think this is something that I was missing out on” (Student 1).

#### Publication Developed Skills and Improved Career Opportunities

Many participants discussed the benefits of publishing in relation to student’s communication skills. Students and graduates reported gaining skills in drafting and revising manuscripts and responding to reviewer feedback; “I submitted the research to an Australian journal and that review and appraising process was …really important” (Graduate 2). Others reported on the value of meeting colleagues in the research community by presenting at conferences. One student shared that they were “…able to meet people that [they] cited in [their] paper” (Student 9). Some graduates also noted the value of the skills gained for their later career, with one stating they were able to “…hit the ground running with skills from ILP” (Graduate 1) and produce research outputs beyond their ILP/Honours project as a clinician. Of the graduates who published their ILP/Honours project findings, most reported that these outputs “…puts (sic) you ahead of other people from other medical schools” (Graduate 6). Supervisors also commented on the advantage a publication gives students when applying for future training opportunities; “…most of my students actually ended up getting publications. And when they apply for scholarships, or going to specialty programs, that actually helps…” (Supervisor 2).

#### Factors Impacting Publication Success

A variety of factors were reported to influence publication success. Both students and graduates reported the need for supervisor and faculty support. One student commented about their supervisor; “He pushed me a lot to get publications and get an outcome from that, which is something I really appreciate now” (Student 5). Another observation was that team dynamics could either support or hinder student publications. For example, two students worked together and contributed to each other’s papers; “we (two students) can share first authorship of the papers that we published” (Graduate 12). By contrast, team dynamics hindered another student publication; “I did my data analysis. And then the other person did their own data analysis….when I asked…will I be on the paper they were like because the other person did their own data analysis, we’re not sure” (Student 2).

Another factor in publication success was the time and motivation required, continuing beyond the research year. Some supervisors commented that the 9 months allocated to the student research was not sufficient to produce peer-review papers. One supervisor stated that “…the publications happen after they leave…” (Supervisor 2). Student motivation was critical to continue their involvement in the long process of writing reviewing and submitting while they were back in full-time clinical studies. One student who did not publish their work acknowledged; “… they [the research team] included me in one of the emails, but I didn't really like follow up” (Student 3).

## Discussion

This research explores publication outputs and experiences in a unique, dedicated research program in the 4th year of an undergraduate medical program in Australia. The findings demonstrate the need for focussed research time in a medical program to enable students to produce high-quality research outputs. At UNSW by 2015, 10 years after the program was first implemented, 50.2% of all student projects resulted in a publication. Out of these, 49.4% of publications had students as first authors, and the majority of outputs were original articles published in Q1 journals. Overall, support, time, and motivation following the research year appeared to be a crucial determinant of publication success.

The ILP/honours year at UNSW Medicine provides students with a dedicated block of time—9 months—to focus on a research project. The value of focussed research time, both for skill development and project outputs, has been reported previously. For example, Duke University has a mandatory research program of 10–12 months during the 3rd year of a 4-year degree with an anecdotal report that “nearly two-thirds” of their students publish at least one article from their research [20]. By contrast, shorter programs between 6 and 20 weeks in length have publication rates below 30%. [21, 22]. Student first authorship is also dependent on time spent on their research; a report from Mayo Medical School demonstrates that when the research program was shortened from 21 to 17/18 weeks, the publication rate was unaffected but the proportion of student first authors decreased from 35 to 18% [23]. Our findings suggest that the 9 month dedicated research time is a strong contributor both to high publication rates and high first author rates.

A further finding of this study was the student time, commitment, and motivation needed beyond the research year for students to publish their findings. The median lag time from the research year to the year of publication was 2 years. Reports from other research programs also comment on the time that it takes after research is completed for students to publish their findings [4, 24]. Student motivation and supervisor support for a sustained period after the project is completed are both critical to enable students to complete and submit a manuscript or conference presentation. Notably, students who negotiated their own research project were more likely to publish than students who had a project allocated to them. Our previous work identified that “Meaningfulness and experience” and “Supervision and support” were key factors that promoted a high rate of student satisfaction amongst ILP/Hon students at UNSW [17]. This is supported by the current study. Students who are supported to negotiate and implement their own research project are more likely to find their projects meaningful and spend the time needed to successfully submit a manuscript through to acceptance in a high-quality peer-reviewed journal.

These findings also suggest that the research program at UNSW has developed and matured over time to better support publication and dissemination of student project findings. A significant increase in the percentage of students who were able to publish their findings was found over time from 2007 to 2015. Previous studies have commented on the time needed for supervisors to develop experience to support student research [22] and the time needed for faculty staff to develop the necessary support structures [25]. A recent report from the University of Pittsburgh, with a longitudinal mandatory program, showed a similar increase in publication rates from 2006 to 2012, with comments on the importance of developing faculty experience over time to support students implementing their own projects [15]. The findings in this study and others highlight that time spent building administrative and supervisor experience is another important contributor to the high rate of quality student publications.

### Strengths and Limitations of the Study

A major strength of this study is that we have used quantitative data on student publications together with qualitative data from interviews with students, supervisors, and graduates to document outputs and explore factors that promote success in disseminating research findings. Using a wide range of databases to conduct the publication audit for three cohorts enabled us to capture changes over time and avoid limitations associated with relying on self-reporting of publications or survey responses. One limitation was that some publications not directly related to the student project may have been included in the audit. However, including both student and supervisor, as well as a title related to the research project, in inclusion criteria, minimised errors of this nature. The sample of participants in the interviews was not representative. An open invitation was emailed by the administrative staff to all supervisors, recent graduates, and year 6 students, and participants willing to participate were recruited. Although the interviewees only represent a convenient sample of these groups, participants were diverse. For example, interviewees had a wide range of project types, and students, graduates, and supervisors with varying experiences were recruited including those who had published and those who had not.

## Conclusion

The time invested into the research program at UNSW has been shown to produce high publication rates and high-quality tangible research outputs. Participants reported that the research skills learned were valuable and that publication had a positive impact on career development. The findings suggest that an investment of quarantined time is warranted to provide medical students with the skills required to practice evidence-based medicine, as well as contribute to scholarly research. Supporting students to negotiate projects has a positive impact on publication success. Lastly, routine thorough documentation of student publications from medical research programs would be a valuable inclusion in any future evaluations.
